# *In Silico* Study of Coumarins and Quinolines Derivatives as Potent Inhibitors of SARS-CoV-2 Main Protease

**DOI:** 10.3389/fchem.2020.595097

**Published:** 2021-02-08

**Authors:** Osvaldo Yañez, Manuel Isaías Osorio, Eugenio Uriarte, Carlos Areche, William Tiznado, José M. Pérez-Donoso, Olimpo García-Beltrán, Fernando González-Nilo

**Affiliations:** ^1^ Computational and Theoretical Chemistry Group, Departamento de Ciencias Químicas, Facultad de Ciencias Exactas, Universidad Andres Bello, Santiago, Chile; ^2^ Center for Bioinformatics and Integrative Biology (CBIB), Facultad de Ciencias de la Vida, Universidad Andres Bello, Santiago, Chile; ^3^ Center of New Drugs for Hypertension (CENDHY), Santiago, Chile; ^4^ Facultad de Medicina, Universidad Diego Portales, Santiago, Chile; ^5^ Departamento Química Orgánica, Facultad de Farmacia, Universidad de Santiago de Compostela, Santiago de Compostela, Spain; ^6^ Instituto de Ciencias Químicas Aplicadas, Universidad Autónoma de Chile, Santiago de Chile, Chile; ^7^ Departamento de Química, Facultad de Ciencias, Universidad de Chile, Santiago, Chile; ^8^ Facultad de Ciencias Naturales y Matemáticas, Universidad de Ibagué, Ibagué, Colombia

**Keywords:** SARS-CoV-2, coumarins, quinolines, protease, molecular dynamics

## Abstract

The pandemic that started in Wuhan (China) in 2019 has caused a large number of deaths, and infected people around the world due to the absence of effective therapy against coronavirus 2 of the severe acute respiratory syndrome (SARS-CoV-2). Viral maturation requires the activity of the main viral protease (M^pro^), so its inhibition stops the progress of the disease. To evaluate possible inhibitors, a computational model of the SARS-CoV-2 enzyme M^pro^ was constructed in complex with 26 synthetic ligands derived from coumarins and quinolines. Analysis of simulations of molecular dynamics and molecular docking of the models show a high affinity for the enzyme (*∆E*_binding_ between −5.1 and 7.1 kcal mol^−1^). The six compounds with the highest affinity show *K*_*d*_ between 6.26 × 10^–6^ and 17.2 × 10^–6^, with binding affinity between −20 and −25 kcal mol^−1^, with ligand efficiency less than 0.3 associated with possible inhibitory candidates. In addition to the high affinity of these compounds for SARS-CoV-2 M^pro^, low toxicity is expected considering the Lipinski, Veber and Pfizer rules. Therefore, this novel study provides candidate inhibitors that would allow experimental studies which can lead to the development of new treatments for SARS-CoV-2.

## Introduction

In recent years, different viruses have emerged in around of the world. These diseases are a generation of respiratory diseases in infected patients, also due to the rapid dissemination of the diseases. These kinds of viruses include the severe acute respiratory syndrome coronavirus (SARS-CoV), Middle East respiratory syndrome coronavirus (MERS-CoV), avian influenza A/H_7_N_9_ and H_5_N_1_ viruses, and Nipah virus (Yuen et al., 1998; Peiris et al., 2003; MacNeil and Rollin, 2012; Marsh and Wang, 2012; To et al., 2012; To et al., 2013; Zaki et al., 2012; Chan et al., 2015). The capacity of these viruses to evolve and infect humans has been associated with the close interaction occurring between human populations and different animal species in markets of densely populated areas (Chan et al., 2015). In December 2019, cases of atypical pneumonia began to be observed in the city of Wuhan (China) (Lu et al., 2020; Zhu et al., 2020). By January 2020, the etiological agent was classified as a new member the of family Coronaviridae and genus β-coronavirus (2019-nCoV) that differ from SARS-CoV and MERS-CoV. The genome of 2019-nCoV shares an 82% sequence identify to SARS-CoV (Elfiky, 2020; Hui et al., 2020; Rothan and Byrareddy, 2020; World Health Organization, 2020). As a matter of fact, its genome has high similarity with the genome of a bat coronavirus (96.2% identity), which has allowed the virus to be associated with a zoonotic origin (Lu et al., 2020; Wu et al., 2020; Zhou et al., 2020). According to the International Committee Virus Taxonomy the new β-coronavirus was called severe acute respiratory syndrome coronavirus 2 (SARS-CoV-2) and on February 11 the set of symptoms associated with this new virus was designated as COVID-19 by the World Health Organization (WHO) (Zhang et al., 2020b). The evolution of infections at a global level increased as the number of cases and deaths, with the most affected countries being the USA, India, Brazil, Russia, France and United Kingdom (COVID-19 Map - Johns Hopkins Coronavirus Resource Center, 2020), which together have presented more than 50% of the global cases in more than 180 countries, declaring it a pandemic on March 11 (WHO Director-General’s opening remarks at the media briefing on COVID-19–20 March, 2020).

Consequently, developed and developing countries are working on the generation of vaccines or antivirals find a solution in the short or medium term. In this context, many governments, medical institutions, and scientists have tried various treatments used for other diseases with promising but so far inconclusive results. These treatments include Chloroquine, Hydroxychloroquine, Camostat, Nafamostat, Umifenovir, Tenofovir, Ramdesidir, Sofosbuvir, Galidesivir, Lopinavir, and indinavir, which are used to treat other diseases but have shown a degree of inhibitory activity of SARS-CoV-2 (Chu et al., 2004; Sissoko et al., 2016; Yamamoto et al., 2016; Mulangu et al., 2019; Cortegiani et al., 2020; Deng et al., 2020; Elfiky, 2020; Grein et al., 2020; Hirota et al., 2020; Hoffmann et al., 2020). Each of these compounds has different modes of action and targets such as antiviral drug (RNA-dependent RNA polymerase (RdRp), viral proteases and membrane fusion clathrin-mediated endocytosis (CME), antimalarial drug (elevation of the endosomal pH and ACE2) and serine protease inhibitor (TMPRSS2) (Chu et al., 2004; Sissoko et al., 2016; Yamamoto et al., 2016; Mulangu et al., 2019; Cortegiani et al., 2020; Deng et al., 2020; Grein et al., 2020; Hirota et al., 2020; Hoffmann et al., 2020; McKee et al., 2020).

Like all other coronaviruses, SARS-CoV-2 is composed by single-stranded RNA as their genetic material with an approximate length of 29,891 nucleotides and a 5′-cap structure and 3′-poly-A tail, encoding 9,860 amino acids (Chan et al., 2020). This RNA encodes both the structural and non-structural proteins of the virus. Among the structural proteins, there is the Spike (S) (present in all coronaviruses), Nucleocapsid (N), Matrix (M), and Envelope (E) (Chan et al., 2020; Walls et al., 2020; Wrapp et al., 2020). Proteases and RNA-dependent RNA polymerase constitute the non-structural proteins of the virus. The genome contains at least six open reading frames (ORFs), the first of these ORF occupies about 60% of the length of the genome and translates two polyproteins known as pp1a and pp1ab, which are processed by the main protease (M^pro^, also called 3CL^pro^) and papain-like proteases (PLPs) (Jin et al., 2020a; Zhang et al., 2020b; Chen et al., 2020). Consequently, inhibiting M^pro^ activity blocks virus replication and thus affects the life cycle of SARS-CoV-2. Compounds derived from coumarins and quinoline have been tested against various viruses (McKee et al., 2020; Mishra et al., 2020). Quinolines have recently been used in experimental treatments for SARS-CoV-2 infected persons in several countries (Arshad et al., 2020; Lopez et al., 2020; Mori et al., 2020) and it has been proposed that coumarins inhibit the replication of several viruses including influenza (Pavurala et al., 2018), HIV (Jesumoroti et al., 2019), Dengue (Coulerie et al., 2013), Chikungunya (Hwu et al., 2019), hepatitis (Hwu et al., 2008) and filoviruses (EBOLA, Marburgvirus (MARV) and Cuevavirus) (Liu et al., 2019).

In this study, we evaluated twenty-sixth molecules derived from coumarins and quinolines as promising SARS-CoV-2 M^pro^ inhibitors, and so, by using computational biochemistry protocols we tried to find the most appropriate molecules that can act as potential anti-SARS-CoV-2 activity drugs. Six of the compounds evaluated are highlighted, which are CTR9, 7HC6, CTR6, 7HC5, 7HC3 and 8HQ6. We performed molecular docking (rigid), efficiency calculations of ligands, pharmacological and toxicological property predictions (ADMET), and molecular dynamics simulations (MD) simulations, together with MM-GBSA binding free energy predictions to identify the binding characteristics for identifying the inhibitors of SARS-CoV-2 M^pro^.

## Computational Methods

### Compounds Set

**FIGURE 1:**
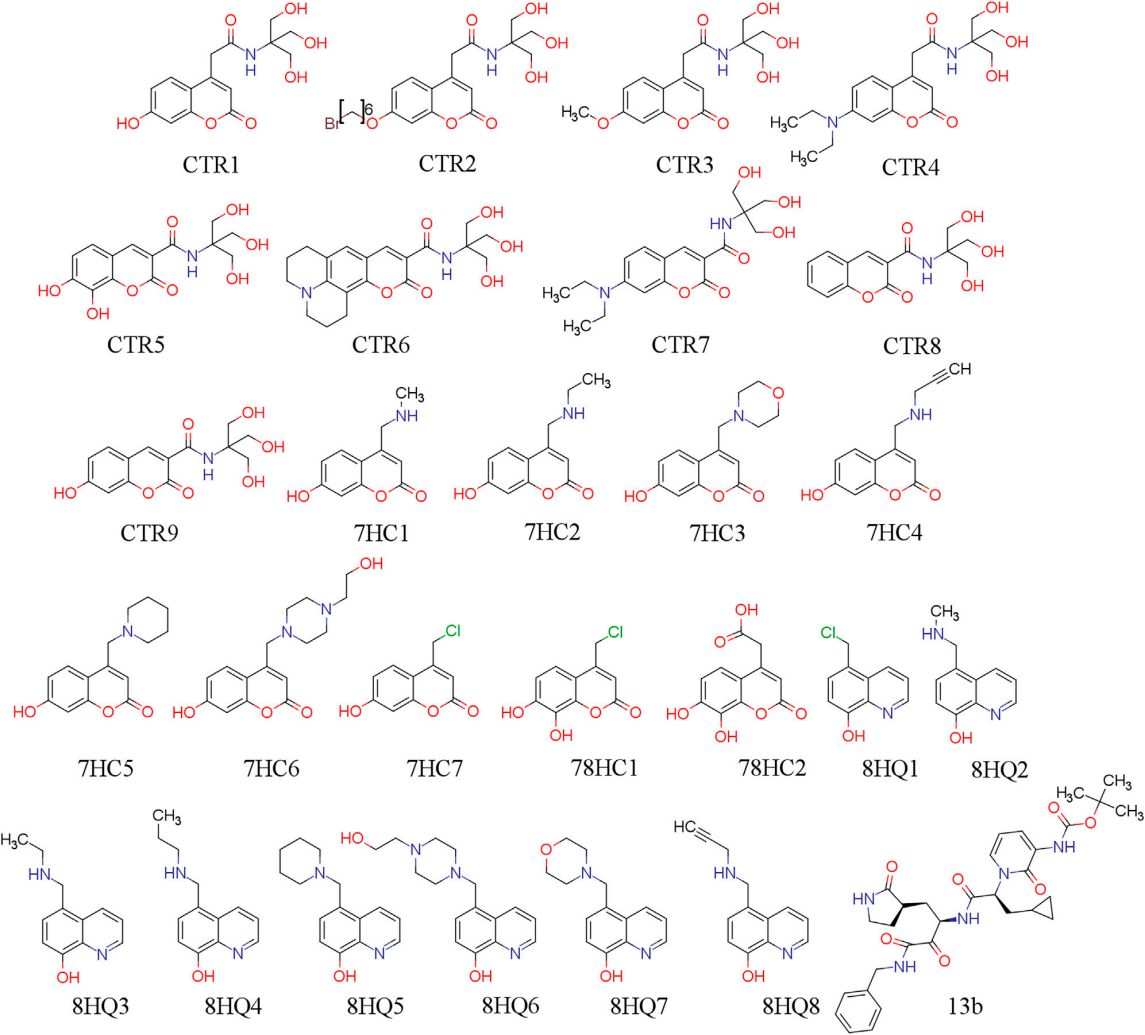
2D chemical structure of ligand under study.

In this work, we use twenty-sixth ligands selected for their possible capability to inhibit SARS-CoV-2 protease M^pro^. The coumarins and quinolines derivatives were extracted from two compound series that have been synthesized by the laboratories of applied chemistry of the Universidad de Ibagué, in Ibague-Colombia. The work was based on strategies that the authors have reported in the literature (García-Beltrán et al., 2013; Mena et al., 2015; Aguirre et al., 2017; Garćia-Beltran et al., 2017). 13b is our reference molecule and was obtained from the Protein Data Bank (PDB) (Zhang et al., 2020b) (PDB id: 6Y2F). These molecules were designed *in silico* and evaluated using docking methodologies and physicochemical and pharmacokinetic descriptors, and to predict ADME parameters. Their chemical structures are shown in Figure 1. Their molecular conformations were optimized using PM6-D3H4 semi-empirical method (Stewart, 2007; Řezáč and Hobza, 2012) as implemented in MOPAC2016 (Stewart, 2016) software. The optimized molecules were used for molecular docking simulations in order to study the interactions established by these compounds in the SARS-CoV-2 M^pro^ pocket.

### Molecular Docking

We made molecular docking analyses to examine the potential binding modes of ligands to the main protease M^pro^ of SARS-CoV-2, as potential inhibitors. Then, based on structural information obtained from the crystal structures of M^pro^ in complex with other ligands we established the binding site of the proposed inhibitors for SARS-CoV-2 M^pro^. In addition to delimit the binding region of possible inhibitors to M^pro^ (Dai et al., 2020; Jin et al., 2020a; ul Qamar, 2020; Xu et al., 2020; Zhang et al., 2020a, Zhang et al., 2020b). We established the key residues for catalysis according to experimental and theoretical data. Finally, we used AutoDock (v 4.2.1) and AutoDock Vina (Trott and Olson, 2010) for all dockings in this study. The initial 3D inhibitors structures were drawn using Discovery Studio (Dassault Systèmes BIOVIA, 2017) 3.1 (Accelrys, CA) which were optimized (considering the RMS gradient of 0.001 kcal/mol) using the PM6-D3H4 semi-empirical method (Stewart, 2007; Řezáč and Hobza, 2012) implemented in the MOPAC2016 (Stewart, 2016) software. PM6-D3H4 introduces dispersion and hydrogen-bonded corrections to the PM6 method. The ligand files were prepared using the AutoDockTools package (Sanner, 1999) provided by AutoDock through accepting all rotatable bonds; moreover, the atomic charges are computed toward the PM6-D3H4 procedure, and non-polar hydrogen atoms are merged. The semi-empirical method has shown to increase significantly docking accuracy and cluster population of the most accurate docking (Bikadi and Hazai, 2009; Fanfrlík et al., 2010; Hou et al., 2013). The crystal structure of SARS-CoV-2 M^pro^ (PDB Code: 6YB7, https://www.rcsb.org/structure/6YB7), was downloaded from the PDB (Berman et al., 2000). The resolution of the retrieved structure was 1.25 Å. The SARS-CoV-2 M^pro^ was treated with the Schrödinger's (Schrödinger, 2020) Protein Preparation Wizard (Madhavi Sastry et al., 2013); polar hydrogen atoms were added, non-polar hydrogen atoms were merged, and charges were assigned. Docking was treated as rigid and carried out using the empirical free energy function and the Lamarckian Genetic Algorithm provided by AutoDock Vina (Morris et al., 1998). The docking grid dimensions were 30.75 × 30.75 × 30.75 Å, making the binding pocket of SARS-CoV-2 M^pro^ the center of mass between amino-acid residues (Cys145 and His41) of the catalytic site. All other parameters were set as defined by default through AutoDock Vina. Dockings were repeated 50 times with space search exhaustiveness set to 100. The best interaction binding energy (kcal·mol^−1^) was selected for evaluation and analyzed according to the potential intermolecular interactions (protein/ligand), such as hydrogen bonding, hydrophobic interactions and the cation–π, π–π stacking. Docking results 3D representations were used. VMD molecular graphics system (Humphrey et al., 1996).

### Ligand Efficiency Approach

Ligand efficiency (*LE*) calculations were performed using one parameter $K_{d}$. The $K_{d}$ parameter corresponds to the dissociation constant between a ligand/protein, and their value indicates the bond strength between the ligand/protein (Abad-Zapatero, 2007, Abad-Zapatero, 2013; Abad-Zapatero et al., 2010). Low values indicate strong binding of the molecule to the protein. $K_{d}$ calculations were done using the following equations:

$$\Delta G^{0}=-2.303RT\log\left(K_{d}\right)$$ (1)

$$K_{d}=10^{\frac{\Delta G^{0}}{2.303RT}}$$ (2)

where $\Delta G^{0}$ corresponds to binding energy (kcal mol^−1^) obtained from docking experiments, *R* is the gas constant whose value is 1.987207 cal/mol K and *T* is the temperature in degrees Kelvin. At standard conditions of aqueous solution at 298.15 K, neutral pH and remaining concentrations of 1 M. The ligand efficiency (*LE*) allows us to compare molecules according to their average binding energy (Reynolds et al., 2008; Abad-Zapatero, 2013). Thus, it determined as the ratio of binding energy per non-hydrogen atom, as follows (Abad-Zapatero, 2007, Abad-Zapatero, 2013; Abad-Zapatero et al., 2010; Cavalluzzi et al., 2017):

$$LE=-\frac{2.303RT}{HAC}\log\left(K_{d}\right)$$ (3)

where $K_{d}$ is obtained from eq. (2) and *HAC* denotes the heavy atom count (i.e., number of non-hydrogen atoms) in a ligand.

### ADMET Properties

The purpose of calculating ADMET profiles is to supply, with reasonable accuracy, a preliminary prediction of the *in vivo* behavior of a compound to assess its potential to become a drug (Yu and Adedoyin, 2003). The molecules used in this study were submitted to the calculation of their absorption, distribution, metabolism, excretion and toxicological properties (ADMET). Also, the physicochemical properties such as molecular hydrogen bond acceptor (*HBA*), hydrogen bond donor (*HBD*), molecular weight (*MW*), topological polar surface area (*TPSA*), rotatable bond count (*RB*) and octanol/water partition coefficient (*LogP*) were calculated using SwissADME webserver (Daina et al., 2017). Compound toxicological properties were analyzed taking into account the Lipinski, Veber and Pfizer toxicity empirical rules, see Table 1. (MacLeod-Carey et al., 2020).

**TABLE 1:** Empirical rules for predicting oral availability and toxicity of a compound.

Properties	Oral availability	Toxicity	Pfizer 3/75 rules
	Lipinski rules	Veber rules	
*MW*	≤500	–	–
*LogP*	≤5	–	≤3
*HBA*	≤10	–	–
*HBD*	≤5	–	–
*TPSA*	–	≤140	≥75
*RB*	–	≤10	−

*MW*: Molecular weight, *LogP*: octanol/water partition coefficient, *HBA*: Hydrogen Bond Acceptor, *HBD*: Hydrogen Bond Donor, *TPSA*: Topological Polar Surface Area and *RB*: Rotatable Bond.

### Molecular Dynamics Simulations

MDs calculations were performed for the lowest six binding energy docking and the compound 13b, which is our reference ligand. These calculations were also obtained from Protein Data Bank (PDB id: 6Y2E). The ligands were bound to SARS-CoV-2 M^pro^ protein (PDB ID:6YB7) in aqueous solutions with an explicit solvent TIP3P water model (Neria et al., 1996) (≈16.000 water molecules). Protonation states of ionizable residues corresponding to pH 7.0 were determined by H++ web interface for computes pK values of ionizable groups in macromolecules and adds missing hydrogen atoms according to the specified pH of the environment (Anandakrishnan et al., 2012). Besides, NaCl ions were modeled to neutralize the systems and maintain an ionic concentration of 0.15 mol/L. The compounds were parameterized by GAFF Force Field for organic molecules. (Wang et al., 2004; ÖzpInar et al., 2010), using the Antechamber module in AmberTools18 with AM1-BCC charges, (Jakalian et al., 2000). The protein structures were modeled with the force field ff14SB. (Salomon-Ferrer et al., 2013). The simulations were carried out using a standard MD protocol: (I) Minimization and structural relaxation of water molecules with 2000 steps of minimization (downward step) and MD simulation with an NPT (300 K) assembly by 1,000 ps using harmonic restrictions of 10 kcal molÅ^−2^ for protein and ligand; (II) minimization of the complete structure considering 2000 downstream minimization steps and 6,500 steps of conjugate gradient minimization; (III) the minimized systems were progressively heated to 300 K, with harmonic restrictions of 10 kcal mol Å^−2^ in the carbon skeleton and ligand during 0.5 ns; (IV) the system was then balanced by 0.5 ns maintaining the restrictions and then by 5 ns without restrictions to 300 K in a canonical assembly (NVT); and (5) finally, a production dynamic was carried out with an isothermal isobaric assembly (NPT) without restrictions for 200 ns at 310 K and 1 atm with a temporary passage of 2 fs. In the MD simulation, the temperature was controlled by the Langevin dynamics with a collision frequency of 1 ps^−1^ (NVT) and the pressure with the Berendsen barostat (NPT). Besides, the Particle Mesh Ewald (PME) method with a cut-off value of 10 Å was used to treat nonbonding and long-range electrostatic interactions. All MD simulation calculations were performed using the AMBER-GPU Implementations18 (Mermelstein et al., 2018). Molecular visualization of the systems and MD trajectory analysis was carried out with the VMD software package (Humphrey et al., 1996).

### Free Energy Calculation

The molecular MM/GBSA method was employed to estimate the binding free energy of the protease-ligand complexes. For calculations from a total of 200 ns of MD, the last 50 ns were extracted for analysis, and the explicit water molecules and ions were removed. The MM/GBSA analysis was performed on three subsets of each system: the protein alone, the ligand alone, and the complex (protein-ligand). For each of these subsets, the total free energy ($\Delta G_{\text{tot}}$) was calculated as follows:

$$\Delta G_{\text{tot}}=H_{\text{MM}}+G_{\text{solv}}-T\Delta S_{\text{conf}}$$ (4)

where $H_{\text{MM}}$ is the bonded and Lennard–Jones energy terms; $G_{\text{solv}}$ is the polar contribution of solvation energy and non-polar contribution to the solvation energy; *T* is the temperature; and $\Delta S_{\text{conf}}$ corresponds to the conformational entropy (Hayes and Archontis, 2012). Both $H_{\text{MM}}$ and $G_{\text{solv}}$ were calculated using AMBER 18 program with the generalized Born implicit solvent model (Götz et al., 2012; Song et al., 2019). $\Delta G_{\text{tot}}$ was calculated as a linear function of the solvent-accessible surface area, which was calculated with a probe radius of 1.4 Å (Abroshan et al., 2010). The binding free energy of SARS-CoV-2 M^pro^ and ligand complexes ($\Delta G_{\text{bind}}$) were calculated by the difference where G_tot values are the averages over the simulation.

$$\Delta G_{\text{bind}}=G_{\text{tot}}\left(\text{complex}\right)-G_{\text{tot}}\left(\text{protein}\right)-G_{\text{tot}}\left(\text{ligand}\right)$$ (5)

### Non-Covalent Interactions

The principal cluster of main component analysis of trajectory were analyzed with the non-covalent interaction index (NCI) (Johnson et al., 2010; Contreras-García et al., 2011) using NCIPLOT program (Contreras-García et al., 2011) to identify and map non-covalent interactions, such hydrogen bonds, steric repulsion, and van der Waals interactions, using the promolecular densities ($\rho^{pro}$), computed as the sum of all atomic contributions. The NCI is based on the electron density (*ρ*), its derivatives and the reduced density gradient (*s*). The reduced density gradient is given by:

$$s=\frac{1}{2{\left(3\pi^{2}\right)}^{1/3}}\;\frac{\nabla \rho }{\rho^{4/3}}$$ (6)

These interactions are local and manifest in real space as low-gradient isosurfaces with low densities which are interpreted and colored according to the corresponding values of sign(*λ*_2_)*ρ*. The surfaces are colored on a blue-green-red scale according to the strength and type of interaction. Blue indicates strong attractive interactions, green indicates weak van der Waals interactions, and red indicates a strong non bonded overlap.

## Results and Discussion

### Molecular Docking Analysis

The docking results, which were conducted to estimate the possible binding sites of potential inhibitors on SARS-CoV-2 M^pro^. The genetic material of SARS-CoV-2 expresses multiple proteins (more than 20 proteins), among these proteins the main protease (Mpro) is identified, a molecule similar to 3 chymotrypsin (3CL^pro^) that shows a similarity of 96.1% with the 3CL^pro^ of SARS-CoV. 3CL^pro^ plays a very important role in replication and transcription processes of the virus genome (Hui et al., 2020). Therefore, 3CL^pro^ is a strategic drug target in the inhibition of the SARS-CoV cycle. The protease is active as a homodimer, structured by the dimerization of two protomers designated as monomer A and monomer B, and the catalytic dyad in each protomer is defined by Cys145 and its residues (Zhang et al., 2020b). This has led to the development of multiple studies with experimental and computational approaches in search of possible inhibitors that can effectively block the activity of this protease (Balaramnavar et al., 2020; Dai et al., 2020; Gao et al., 2020; Jin et al., 2020a; Jin et al., 2020b; Ngo et al., 2020; Zhang et al., 2020b). Work with the 3C-like proteinase from SARS coronavirus revealed that the Cys145 residue is key at the active site of 3CLpro (Huang et al., 2004), this advance allowed the mentioned residue to be an attractive target for covalent ligands to bind acting as inhibitors of 3CL^pro^. This amino acidic residue is also a popular target for covalent inhibitors because of its intrinsic reactivity at physiological pH (Cuesta et al., 2020). Tung Ngo et al. report in recent studies that additionally Glu166 residue has a prominent and important role in binding ligands to SARS-CoV-2 M^pro^ (Ngo et al., 2020).

Twenty-seven inhibitors, including the reference ligand 13b were evaluated *in silico* anti-SARS-CoV-2 activity. The results of this study of molecular docking calculations indicate the strong interactions of molecules derived from coumarins and quinolones targets the Cys-His catalytic dyad (Cys145 and His41) in the binding pocket of SARS-CoV-2 M^pro^. The results of the binding of these molecules are presented in Table 2. Meanwhile, the binding to various amino acid residues due to their presence in the conserved region of the active site in all compounds is seen and presents a very important role in enzymatic catalysis.

**TABLE 2:** Molecular docking study between selected ligands and SARS-CoV-2 M^pro^. Intermolecular docking values, presented with their interaction energy (*∆E*_*binding*_), H-bond residues, interacting residues are shown and Ligand efficiency calculation for SARS-CoV-2 M^pro^ complexes.

Compound	Docking results			Ligand Efficiency	
	*∆E*_binding_ (kcal mol^−1^)^a^	H-bonds^b^	Residue interactions^b^	*K*_*d*_	*LE* (kcal mol^−1^)
13b^c^	–7.2	Leu167; Glu166	Arg188; Asn142; Asp187; **Cys145**; Gln189; Glu166; Gly143; His164; His163; **His41**; Leu167; Leu27; Met165; Met49; Phe140; Pro168; Ser144; Thr25; Thr26	5.29 × 10^–6^	0.167
CTR6	–7.1	Met49; Gln189; Glu166; Gln192	Arg188; Asn142; **Cys145**; Gln189; Gln192; Glu166; Gly143; **His41**; Leu167; Met49; Pro168; Thr190; Thr25	6.26 × 10^–6^	0.253
7HC6	–6.7	Glu166; Ser46	Arg188; **Cys145**; Cys44; Gln189; Gln192; Glu166; **His41**; Met165; Met49; Ser46; Thr25; Thr45	12.3 × 10^–6^	0.304
7HC5	–6.6	–	**Cys145**; Glu166; His164; His163; **His41**; Met165; Met49; Phe140; Ser144; Ser305	14.6 × 10^–6^	0.347
CTR9	–6.6	Met165; Asn142	Asn142; **Cys145**; Cys44; Glu166; Gly143; His164; His163; Leu141; Met165; Met49; Phe140; Ser144; Ser46; Thr25; Thr45	14.6 × 10^–6^	0.300
7HC3	–6.6	–	**Cys145**; Glu166; His164; His163; **His41**; Leu141; Met165; Met49; Phe140; Ser144; Ser305	14.6 × 10^–6^	0.347
8HQ6	–6.5	–	**Cys145**; Cys44; Glu166; His164; **His41**; Leu141; Met165; Met49; Phe140; Ser144; Ser305; Thr25	17.2 × 10^–6^	0.309
CTR4	–6.5	His163; Glu166; Met49	Asn142; **Cys145**; Cys44; Glu166; His163; His172; Leu141; Met165; Met49; Phe140; Ser144; Ser305; Ser46; Thr25; Thr45	17.2 × 10^–6^	0.240
CTR5	–6.4	His163; Thr26; Gly143; Thr25	Asn142; **Cys145**; Glu166; Gly143; His163; Leu141; Met165; Phe140; Ser144; Thr24; Thr25; Thr26	20.4 × 10^–6^	0.278
CTR1	–6.3	His41; Glu166; Arg188; Leu27; Thr25	Arg188; Asn142; **Cys145**; Gln189; Gln192; Glu166; Gly143; **His41**; Leu27; Met165; Met49; Thr190; Thr25; Thr26	24.1 × 10^–6^	0.273
CTR3	–6.3	Glu166; His163; Asn142	Asn142; **Cys145**; Cys44; Glu166; His163; His172; Leu141; Met165; Met49; Phe140; Ser144; Ser305; Ser46; Thr25; Thr45	24.1 × 10^–6^	0.262
8HQ5	–6.3	Gln189	**Cys145**; Gln189; Glu166; His163; Met165; Met49; Phe140; Ser144; Ser305	24.1 × 10^–6^	0.350
8HQ7	–6.2	–	**Cys145**; Gln189; Glu166; His163; Met165; Met49; Phe140; Ser144; Ser305	28.6 × 10^–6^	0.344
78HC2	–6.1	His163	Asn142; **Cys145**; Gln189; Glu166; His163; Met165; Phe140; Ser144	33.8 × 10^–6^	0.358
CTR7	–6.1	Thr25; Cys44	Arg188; Cys44; Gln189; Gln192; Glu166; **His41**; Leu167; Met165; Met49; Pro168; Ser46; Thr190; Thr25; Thr45	33.8 × 10^–6^	0.234
CTR2	–6.1	Leu141; Thr25; His163; Asn142	Asn142; **Cys145**; Glu166; Gly143; His163; Leu141; Leu27; Met165; Met49; Phe140; Ser144; Ser46; Thr25	33.8 × 10^–6^	0.203
CTR8	–6.0	Gly143	Asn142; **Cys145**; Glu166; Gly143; His163; Leu141; Met165; Phe140; Ser144; Thr25; Thr26	40.1 × 10^–6^	0.285
78HC1	–5.7	–	**Cys145**; Glu166; His163; Leu141; Met165; Phe140; Ser144; Ser305	66.5 × 10^–6^	0.380
7HC2	–5.7	–	**Cys145**; Glu166; His164; His163; Leu141; Met165; Phe140; Ser144; Ser305	66.5 × 10^–6^	0.356
7HC4	–5.7	Phe140; Asn142	Asn142; **Cys145**; Glu166; Gly143; His163; **His41**; Leu141; Leu27; Met165; Phe140; Ser144	66.5 × 10^–6^	0.335
7HC1	–5.6	–	**Cys145**; Glu166; His164; His163; **His41**; Met165; Phe140; Ser144; Ser305	78.6 × 10^–6^	0.373
8HQ4	–5.6	Glu166	Arg188; **Cys145**; Gln189; Glu166; His163; Leu141; Met165; Met49; Phe140; Ser144; Ser305	78.7 × 10^–6^	0.350
7HC7	–5.5	His163; Phe140	**Cys145**; Glu166; His163; Leu141; Met165; Phe140; Ser144	93.2 × 10^–6^	0.392
8HQ8	–5.4	Met165	Asn142; **Cys145**; Glu166; **His41**; Met165; Met49	110 × 10^–6^	0.337
8HQ2	–5.3	Glu166	**Cys145**; Glu166; His163; Met165; Phe140; Ser144; Ser305	130 × 10^–6^	0.378
8HQ3	–5.3	Glu166	**Cys145**; Gln189; Glu166; His163; Met165; Met49; Phe140; Ser144; Ser305	130 × 10^–6^	0.353
8HQ1	–5.1	–	Asn142; Glu166; His164; His163; Leu141; Met165; Phe140; Ser144; Ser305	182 × 10^–6^	0.392

^a^ In each site, the energy was calculated to see which site had the highest degree of union with the ligand.

^b^ The reason of 3 Å was the length of the Hydrogen bond ranges from 2.6 Å to 3.1 Å based on observations from the PDB.

^c^ The ligand 13b is our reference ligand and was obtained from PDB (id: 6Y2E).

His41 and Cys145 residues of the catalytic site are highlighted with bold font.

**FIGURE 2:**
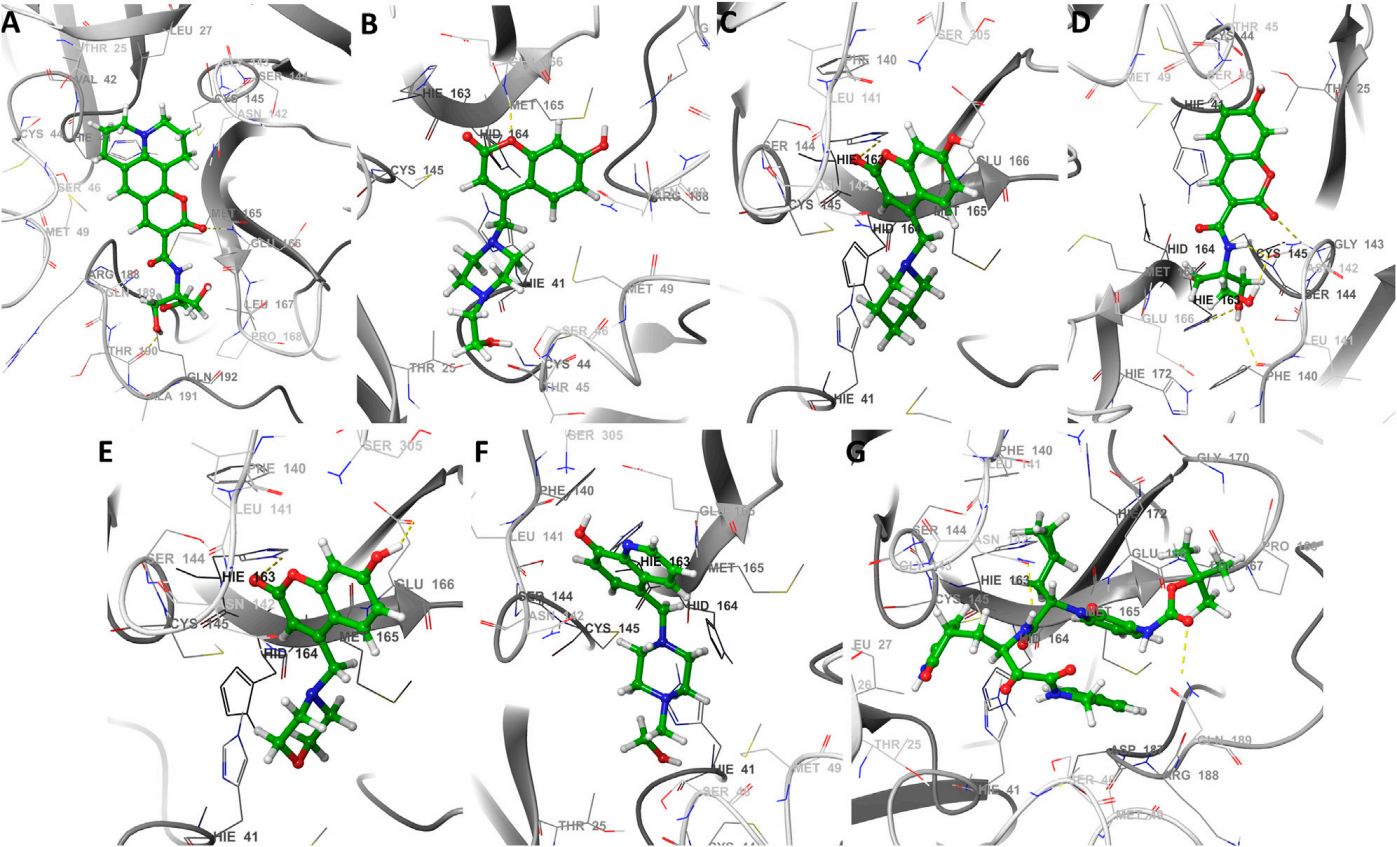
The best seven docking poses of different ligands in SARS-CoV-2 M^pro^ binding pocket. Snapshots of **(A)** CTR6, **(B)** 7HC6, **(C)** 7HC5, **(D)** CTR9, **(E)** 7HC3, **(F)** 8HQ6 and **(G)** 13b during docking simulations. The yellow dotted line usually represents intermolecular interactions, like hydrogen bonds.

Docking results with SARS-CoV-2 M^pro^, indicated that all ligands present binding energies between −5.3 and −7.2 kcal mol^−1^ (Table 2), with a difference of 0.1 kcal mol^−1^ between 13b and CTR6, which means that the level of stability is very similar between these two protease-complex ligands. AutoDock Vina, presents that the ligand 13b attached to the co-crystal, and the re-coupled ligand 13b presents an RMSD value of 3.1 Å, suggesting a partially acceptable value of the coupling method. Furthermore, it shows that the compound CTR6 gives the lowest energy (−7.1 kcal-mol^−1^) in complex with the protease, which is the best score when compared to other docked compounds used in this study. CTR6 gives better score than 7HC6 (−6.7 kcal mol^−1^), 7HC5 (−6.6 kcal mol^−1^), CTR9 (−6.6 kcal mol^−1^), 7HC3 (−6.6 kcal mol^−1^), 8HQ6 (−6.5 kcal mol^−1^) and the other compounds. The coumarins and quinolines derivatives are located inside the protein pocket, by means of electrostatic and hydrophobic interactions with the residues Arg188, Asn142, Asp187, Cys145, Gln189, Glu166, Gly143, His164, His163, His41, Leu167, Leu27, Met165, Met49, Phe140, Pro168, Ser144, Thr25 and Thr26. In relation to 13b, it shows two hydrogen bonding interactions with Leu167 and Glu166 residues. In the case of CTR6, 7HC6 and CTR9 in complex with SARS-CoV-2 M^pro^ (Figure 2 and Table 2) show possible hydrogen-bonding interactions with the residues Ser46, Met49, Asn142, Met165, Glu166, Gln189 and Gln192 (H-donor). This allows us to conclude that the hydrogen bonds and hydrophobic forces, are the majority interactions that dominate these complexes. Interactions between the rest of the compounds and SARS-CoV-2 M^pro^ are reported in Table 2.

### Ligand Efficiency Analysis

The parameters dissociation constant (*K*_*d*_) and ligand efficiency (*LE*) were used to compare the affinity of the molecules studied and SARS-CoV-2 M^pro^. The *K*_*d*_ of a ligand-protease complex, the values shown indicate the strength of the protein-ligand interaction. Very low values are an indicator that the ligand has a very close bond to the protein. LE represents the average bonding energy per non-hydrogen atom, giving standardized values allowing to compare the molecules derived from coumarins and quinolines of different sizes, see Table 2. The six best ligands obtained from the docking exhibit low *K*_*d*_ values, which leads to the conclusion that these complexes are the most stable of the series presented, these ligands are CTR6, 7HC6, 7HC5, CTR9, 7HC3 and 8HQ6, including the reference ligand 13b. The results are coherent with those obtained in the molecular docking, where these complexes according to the values of *∆E*_binding_ showed greater stability. Default tolerable values of *LE* of inhibitor candidate compounds should show *LE* values >0.3 kcal-mol^−1^. According to the values, the compounds 7HC6, 7HC5, CTR9, 7HC3 and 8HQ6 are excellent prospects to be used as SARS-CoV-2 M^pro^ inhibitors.

### Molecular Dynamics Simulation and MM/GBSA Analysis

Molecular dynamics simulations were performed in 200 ns to analyze the steady nature and conformations stability of ligand-SARS-CoV-2 M^pro^ complexes (ligands: CTR9, 7HC6, CTR6, 7HC3, 8HQ6 and 7HC5).

**FIGURE 3:**
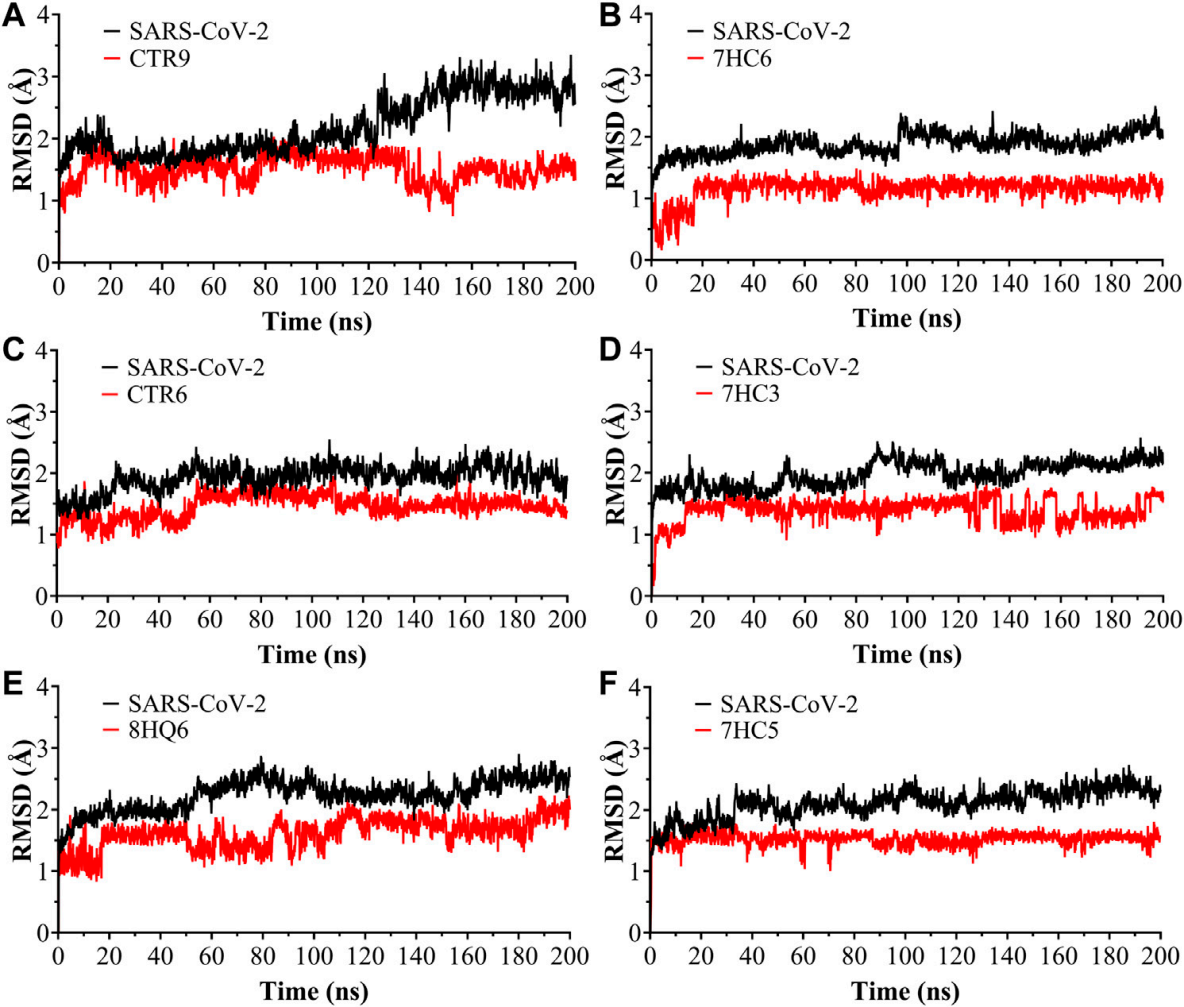
Root Mean Square Deviation (RMSD) as a function of simulated times for the complexes formed between SARS-CoV-2 M^pro^ and **(A)** CTR9, **(B)** 7HC6, **(C)** CTR6, **(D)** 7HC3, **(E)** 8HQ6 and **(F)** 7HC5 molecules.

**FIGURE 4:**
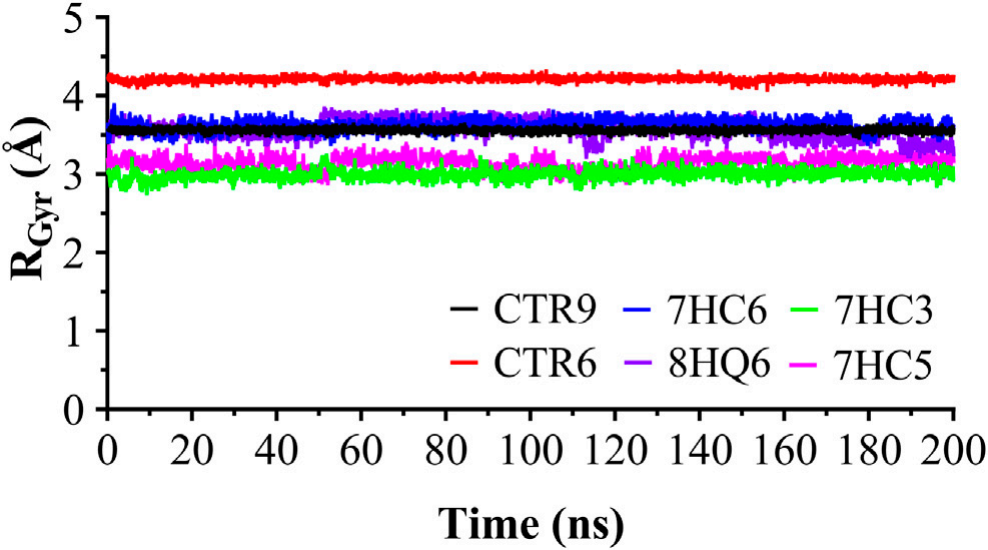
Radius of gyration for the SARS-CoV-2 M^pro^ in complex with CTR9, 7HC6, CTR6, 7HC3, 8HQ6 and 7HC5, during 200 ns simulation time.

The RMSD was used to estimate the stability of protein-ligand systems. RMSD trajectories of SARS-CoV-2 M^pro^-ligand complexes during 200 ns simulation indicated that the complexes formed with the ligands during the DM simulations have a high stability during the simulation time (Figure 3). The structure does not show significant changes, in this case there is an increase of the RMSD until reaching a point in which the values fluctuate around values of 0.5 and 1.8 Å of RMSD. After a Molecular dynamics of 200 ns the structures remain within the parameter that considers the system to be in equilibrium, therefore, no complex suffered structural destabilization during the simulation. Deviations with a maximum difference of 3.0 Å of RMSD (Carugo, 2003) indicate that the system is in equilibrium, situation that is fulfilled for the simulation of the possible SARS-CoV-2 M^pro^ indicating equilibrium states of the ligands within the active site of protease. Also, RMSD curves for 7HC5 and CTR6 are remarkably more stable than those of CTR9, 7HC6, 7HC3 and 8HQ6. To complement the analysis carried out from the RMSD, the study of the Radius of Gyration (R_Gyr_) was carried out for the same runs. From R_Gyr_ analysis Figure 4, we can conclude that the R_Gyr_ of ligands 7HC5 and 7HC3 have values that oscillate in an interval close to 3.0 Å, for the case of CTR9, 8HQ6 and 7HC6 have values fluctuating in an interval close to 3.5 Å and for the CTR6 ligand it has values higher than 4.0 Å. The stable values during the 200 ns simulation for R_Gyr_ indicate that ligand binding at the active site of the protein does not induce major conformational changes in the protein structure.

**FIGURE 5:**
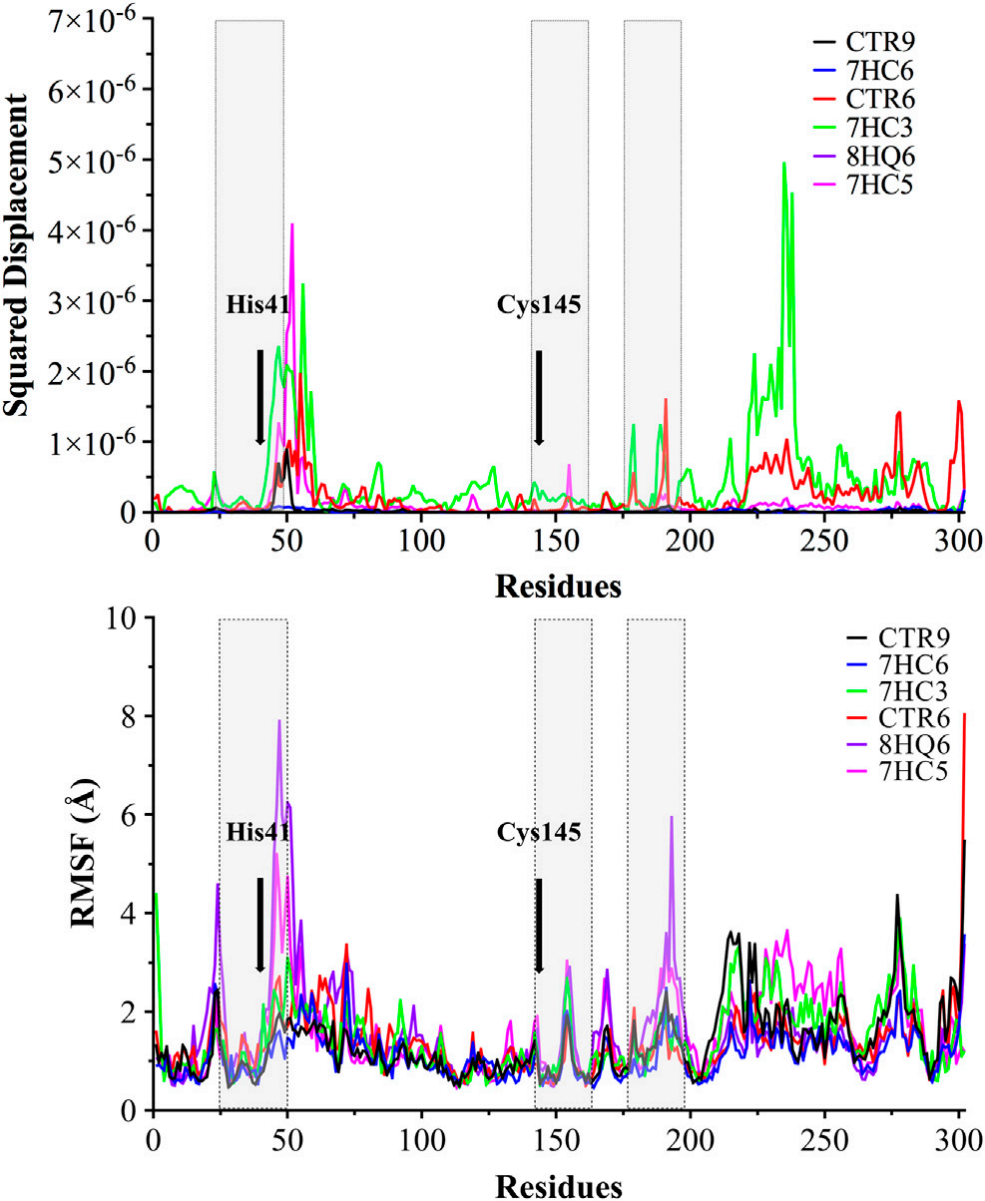
Normal Mode Analysis and RMSF of the α-carbon. A main component analysis was carried out using the last 100 ns trajectories, and the main normal mode of movement was obtained. The displacement was plotted for each residue of SARS-CoV-2 M^pro^ in complex with CTR9, 7HC6, CTR6, 7HC3, 8HQ6 and 7HC5. In grey boxes represented pocket site residues. His41 and Cys145 residues of the catalytic site are highlighted with bold font.

With the purpose of identifying the deviation of the ligand from respect to its initial position and the movement of proteins residues, the Normal Mode Analysis (NMA) and Root Mean Square Fluctuations (RMSF) values were calculated averaging over all the conformations sampled during the last 100 ns simulation. The NMA and RMSF were calculated using the Cα atom of each amino acid residue as a reference and the graph was used to represent the fluctuations in the residue level. NMA plot in Figure 5 shows a similar trend of residue fluctuation profile for the complex with an average NMA of 1.0 × 10^–6^ fluctuations. The two CTR6-protease and 7HC3-protease complexes showed a comparatively higher fluctuation in some residues. This trend in the quadratic displacement figure of the complex suggests that the binding of the six compounds to the protein showed stability and no effect on the flexibility of the protein was observed in the whole range of the simulations. The N-terminus to C-terminus of the proteins normally present great fluctuations and in most cases their movement does not represent importance. As shown in Figure 5, the RMSF graph, all the compounds made the residues present the same fluctuation, except for the two 8HQ6-protease and 7HC5-protease complexes showed a comparatively higher fluctuation in some residues. We consider and from the theoretical point of view, that these ligands present movements in the active site to settle in the best orientation, reason why part of the amino acidic residues fluctuate more than normal. However, other ligands exhibit good behavior and their RMSF values show that they can handle the fluctuations of the residues. On the active site of the protease, the fluctuation values of the main residues (Zhang et al., 2020a) (His41, His163, His164, Phe140 and Cys145) of the six selected molecules were similar among them. The results of the MD simulation indicate that two of the ligands obtained from the coupling analysis (CTR9 and 7HC6) remain close to their initial locations even in uncontrolled simulations, which points to the constitution of stable complexes. From these results it can be clearly deduced that it is likely that the molecules CTR9 and 7HC6 play the same role in inhibiting SARS-CoV-2 M^pro^ as 13b.

**FIGURE 6:**
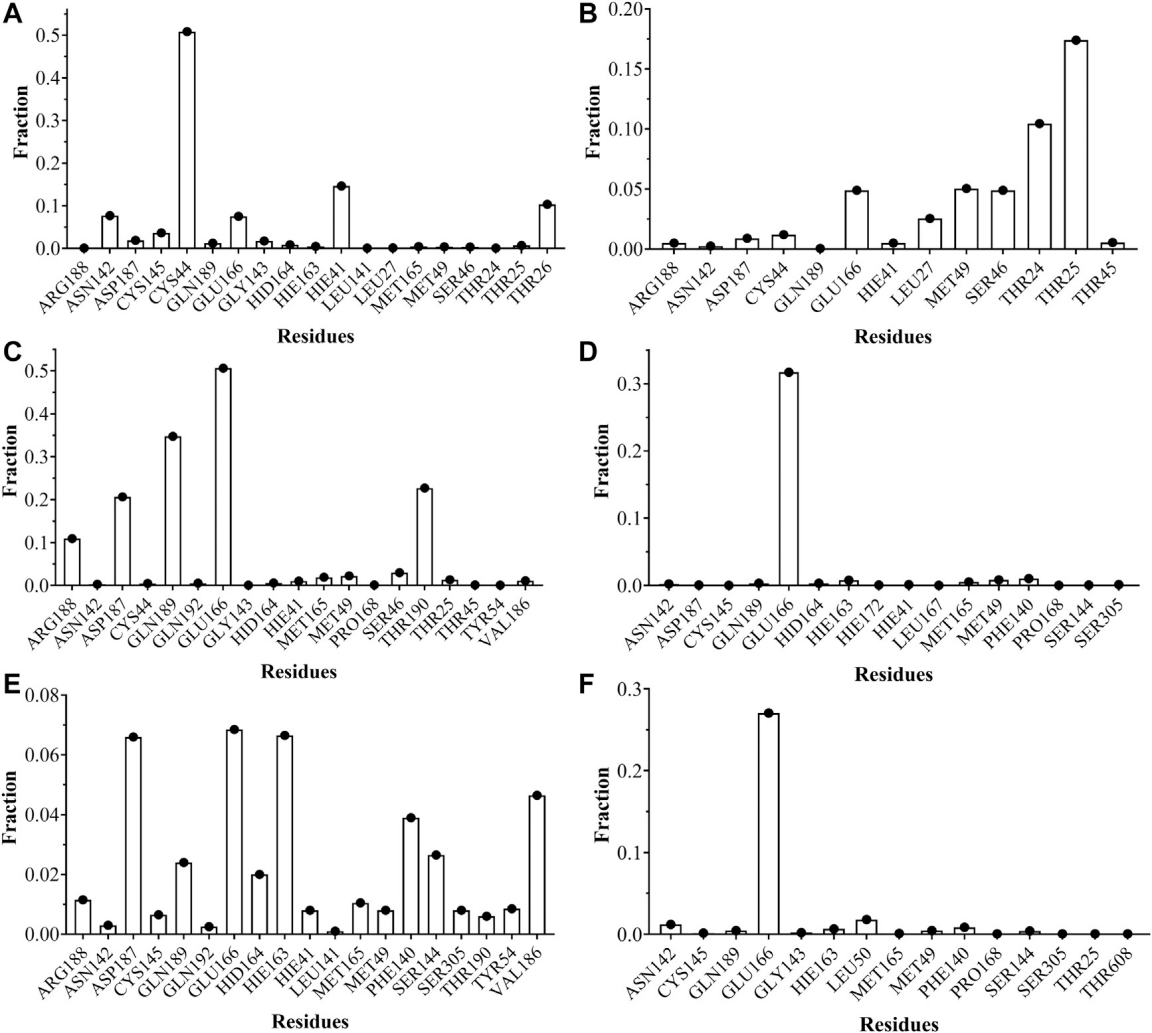
Fraction of intermolecular hydrogen bonds for SARS-CoV-2 M^pro^ interacting with **(A)** CTR9, **(B)** 7HC6, **(C)** CTR6, **(D)** 7HC3, **(E)** 8HQ6 and **(F)** 7HC5. The graph bar shows the most common hydrogen bonds formed between the residues on the pocket and the inhibitors. Values obtained from CPPTRAJ script in AMBER.

**FIGURE 7:**
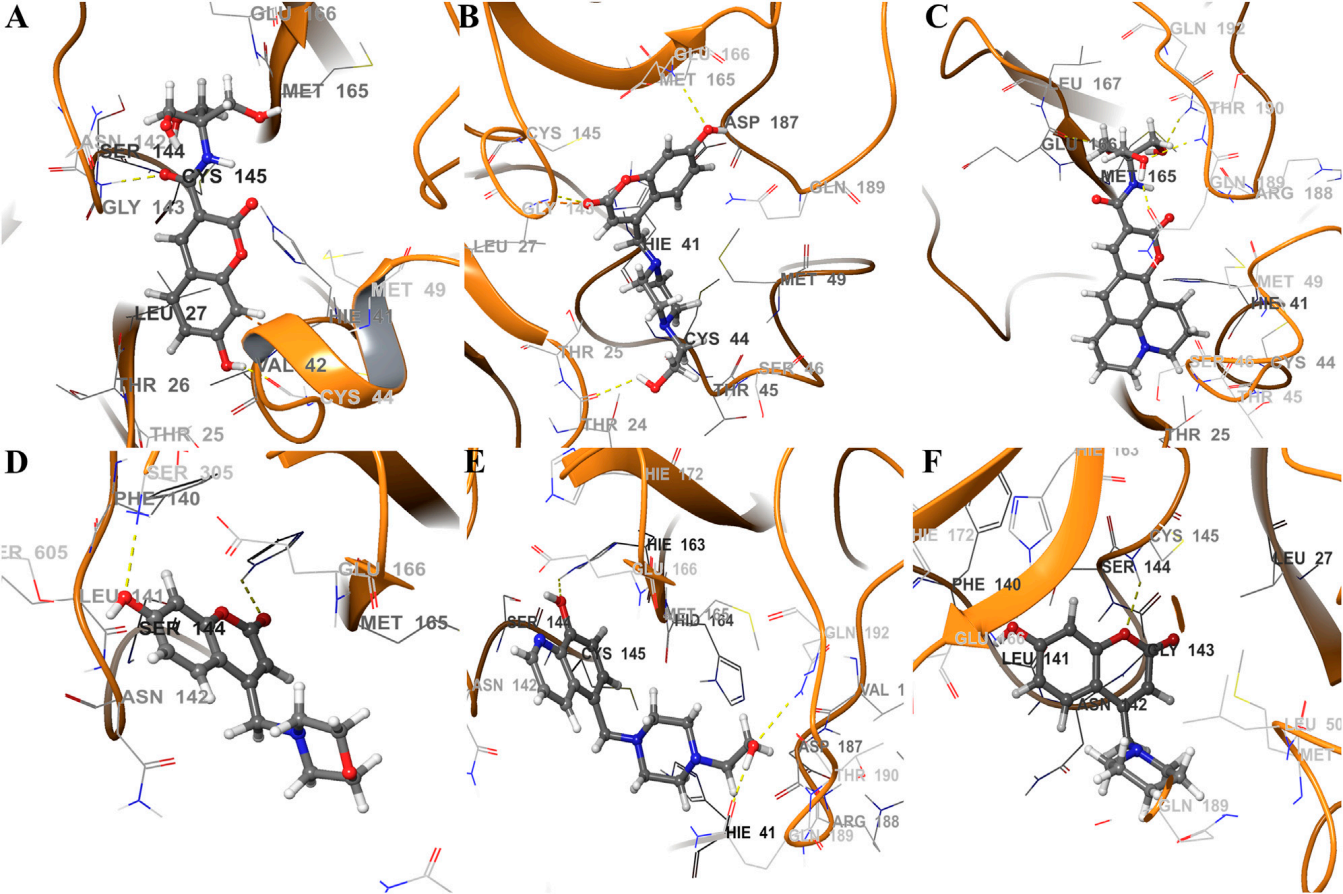
Schematic representations of main component analysis of their respective production run for ligands **(A)** CTR9, **(B)** 7HC6, **(C)** CTR6, **(D)** 7HC3, **(E)** 8HQ6 and **(F)** 7HC5 bound to SARS-CoV-2 M^pro^. The surrounding amino acid residues in the binding pocket of SARS-CoV-2 M^pro^ within 3Å from ligands. The yellow dotted line usually represents intermolecular interactions, like hydrogen bonds.

The analyses of trajectories indicate that during most of the simulation the ligands CTR9, 7HC6, CTR6, 7HC5, 7HC3 and 8HQ6 maintain hydrogen bonds with residues of the active site of SARS-CoV-2 M^pro^. However, the number of hydrogen bridges formed was different for each ligand (Figure 6). CTR9 formed three hydrogen bridges between the residues Cys44, His41 and Thr26, highlighting the participation of the residues Glu166, Cys145, Asn142 and Asp187. 7HC6 formed two hydrogen bridges between the residues Thr24 and Thr25, highlighting the participation of the residues Leu27, Ser46, Met49 and Glu166. In the case of CTR6, three hydrogen bridges between the residues Glu166, Gln189 and Thr190 were determined, highlighting the participation of the residues Arg188 and Ser46. 7HC3 formed one hydrogen bridge with the residue Glu166, highlighting the participation of the residues His163, Met49 and Phe140. Three hydrogen bridges are formed between 8HQ6 and the residues Asp187, Glu166 and His163, highlighting the participation of the residues Gln189, Phe140, Ser144 and Val186. Finally, 7HC5 formed one hydrogen bridge with the Glu166 residue, highlighting the participation of the residues Leu50, Asn142 and Phe140. These residues, see Figures 6 and 7, are consistent with previous theoretical-experimental studies carried out by Dai et al. (Dai et al., 2020), where they detail the interaction that some of the synthesized compounds have with the active site of the protease.

**FIGURE 8:**
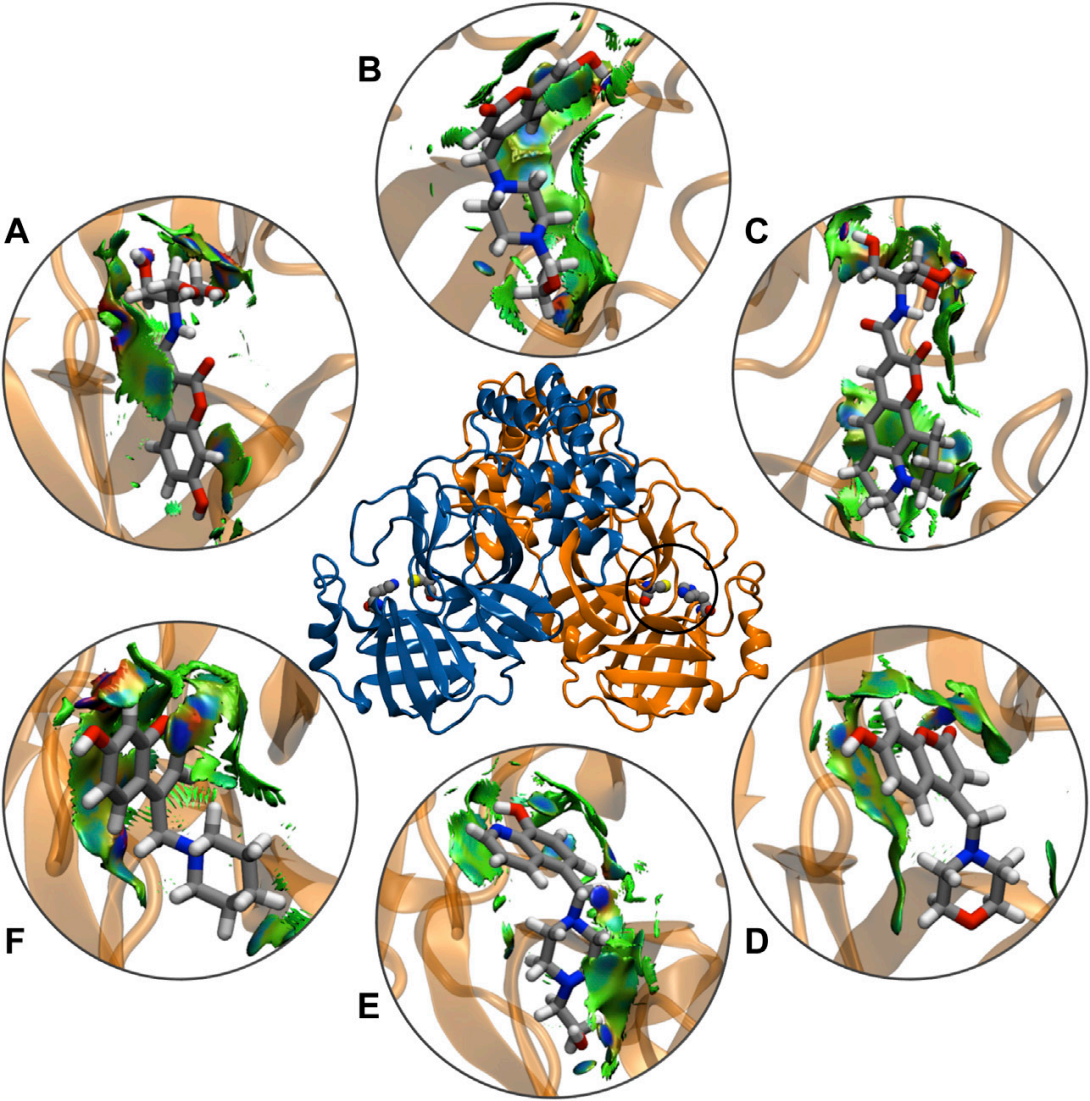
Schematic representations of main component analysis of their respective production run for NCIPLOT isosurface gradient (0.6 au) of ligands **(A)** CTR9, **(B)** 7HC6, **(C)** CTR6, **(D)** 7HC3, **(E)** 8HQ6 and **(F)** 7HC5 on the structure of SARS-CoV-2 M^pro^. The color scale is −2.0 < *ρ* < 2.0 au.

The noncovalent interactions analysis labeled all the hydrogen-bonding interactions in total agreement with the molecular dynamics simulations, providing a qualitative confirmation of these interactions, using a topological and visual analysis of a scalar field related to the electron density (Figure 8). These results suggest that the better inhibitors character is due to direct mechanisms.

Finally, the binding free energy (MM-GBSA) was estimated subsequent to the MD simulation; the last 50 ns for all the complexes and the results are given in Table 3. CTR9 and 7HC6 compounds depicted the lowest binding free energy (−24.8 and −24.7 kcal mol^−1^) with SARS-CoV-2 M^pro^, while the compounds CTR6, 7HC5, 7HC3 and 8HQ6 showed relatively higher binding energy (−24.7, −22.8, −22.6, −20.5 and −20.5 kcal mol^−1^). On the other hand, the reference compound 13b showed the lowest binding free energy (−29.1 kcal mol^−1^) with the SARS-CoV-2 M^pro^ in comparison to compound CTR9, with a slight difference of 4.3 kcal mol^−1^. Although compound 13b has a lowest binding free energy, it presents the problem of breaking the rules of Lipinski and Veber rules, however compound CTR9 does not.

**TABLE 3:** Predicted binding free energies (*∆G*_binding_) calculated from molecular dynamics simulation through the MM/GBSA protocol for SARS-CoV-2 M^pro^ complexes.

Ligand	*∆G*_binding_ (kcal·mol^−1^)
13b^a^	−29.1 ± 0.12
CTR9	−24.8 ± 0.12
7HC6	−24.7 ± 0.07
CTR6	−22.8 ± 0.10
7HC5	−22.6 ± 0.08
7HC3	−20.5 ± 0.11
8HQ6	−20.5 ± 0.11

^a^ The ligand 13b is our reference ligand and was obtained from PDB (id: 6Y2E).

### ADMET Properties

In the search for new drugs, safety is very important and the regulations related to ADMET (Absorption, Distribution, Metabolism, Excretion and Toxicity), most of the time are the cause for a drug to fail. Therefore, it is of utmost importance to identify aspects such as the toxicity of compounds in early stages of development and thus avoid the loss of resources and time. (Sivamani et al., 2012). To evaluate the best ligands as potential anti-SARS-CoV-2 activity drugs; we have calculated some pharmacokinetic properties (Table 4). These results were compared to Veber’s (Veber et al., 2002), Pfizer’s (Hughes et al., 2008) and Lipinski’s rule (Lipinski et al., 2001). in the development of new drugs if a molecule complies only with one of the Lipinski's rule is not an appropriate candidate and it is not relevant to continue with the study, however, by presenting a greater number of rules the probabilities of being a candidate begin to increase and deepen their study. In accordance with Veber's rule, if a compound does not satisfy at least two parameters, it is not a candidate for further development. In addition, Pfizer 3/75 toxicity rules have also been taken into account in this study, concluding that if any of the proposed ligands do not meet the established parameters, then it is not a suitable candidate.

**TABLE 4:** ADME molecular descriptors of compounds designed to inhibit SARS-CoV-2 M^pro^.

Compound	*MW* (g/mol)	*LogP*	*HBA*	*HBD*	*TPSA* (Å^2^)	*RB*
13b	593.67	2.27	7	4	164.70	17
CTR6	388.41	0.94	6	4	123.24	6
7HC6	304.34	0.93	6	2	77.15	4
7HC5	259.3	2.27	4	1	53.68	2
CTR9	309.27	−0.16	7	5	140.23	6
7HC3	261.27	1.40	5	1	62.91	2
8HQ6	287.36	1.16	5	2	59.83	4
CTR4	378.42	0.65	6	4	123.24	10
CTR5	325.27	−0.71	8	6	160.46	6
CTR1	323.3	−0.31	7	5	140.23	7
CTR3	337.32	0.02	7	4	129.23	8
8HQ5	242.32	2.52	3	1	36.36	2
8HQ7	244.29	1.65	4	1	45.59	2
78HC2	236.18	0.62	6	3	107.97	2
CTR7	364.39	0.83	6	4	123.24	9
CTR2	486.35	1.99	7	4	129.23	14
CTR8	293.27	0.31	6	4	120.00	6
78HC1	226.61	1.54	4	2	70.67	1
7HC2	219.24	1.63	4	2	62.47	3
7HC4	229.23	1.61	4	2	62.47	3
7HC1	205.21	1.29	4	2	62.47	2
8HQ4	216.28	2.22	3	2	45.15	4
7HC7	210.61	2.03	3	1	50.44	1
8HQ8	212.25	1.85	3	2	45.15	3
8HQ2	188.23	1.53	3	2	45.15	2
8HQ3	202.25	1.85	3	2	45.15	3
8HQ1	193.63	2.30	2	1	33.12	1

ADME prediction showed that in most cases, all the compounds proposed in this study satisfy with the Veber’s, Pfizer’s and Lipinski’s rule. This suggests that these ligands could be safe molecules for use as anti-SARS-CoV-2 activity drugs. In the case of reference ligand 13b presents a violation of Lipinski’s and Veber’s rule, due to their molecular weight, topological polar surface area and rotatable bond count. The values of these properties are higher than the admissible limit, making this substance fat-soluble which indicates a tendency to be more toxic and less selective to their target. In the case of the six best compounds found in the docking simulations (CTR6, 7HC6, 7HC5, CTR9, 7HC3 and 8HQ6), all of them satisfactorily meet the Veber’s, Pfizer’s and Lipinski’s rule. These compounds represent most promising compounds to molecular dynamics simulation and MM-GBSA.

## Conclusion

This paper predicts that compounds derived from coumarins and quinolines that can be successfully potential drugs to treat viral diseases such as COVID-19. Herein, we used a computational chemistry protocol to identify the ligands most promising candidates that may inhibit main protease of SARS-CoV-2 activity determined by means of this protocol involves of molecular dockings, molecular dynamics simulations, MM-GBSA, NCI and ADMET properties to predict whether these compounds are appropriate to be utilized in an anti-COVID-19 therapy. We identified six compounds (CTR9, 7HC6, CTR6, 7HC5, 7HC3, 8HQ6) that are already synthesized (García-Beltrán et al., 2013; Mena et al., 2015; Aguirre et al., 2017; Garćia-Beltran et al., 2017) with a potential inhibition of main protease of SARS-CoV-2. These compounds might be repurposed against COVID-19. These hits were described as drug-like compounds and showed harmless ADMET properties and may aid in developing and optimizing more efficient and potent COVID-19 inhibitors. Trajectory analysis showed that the studied complexes display structural stability during the MD runs. These results encourage further *in vitro* and *in vivo* investigations and also preventively boost the traditional use of coumarins and quinolines derivatives preventively. We anticipate that the insights obtained in the present study may prove valuable for researching and developing novel anti-COVID-19 therapeutic agents in the future.

## Data Availability Statement

The original contributions presented in the study are included in the article/Supplementary Material, further inquiries can be directed to the corresponding author.

## Author Contributions

OY, OG-B, CA, WT and FG-N contributed to the conception and design of the study; OY, MIO and JPD preformed the theoretical calculations; OY, EU, MO and JP-D organized the database; OY, MO, JP-D, CA and OG-B wrote the first draft of the manuscript; OY, OG-B, and FG-N wrote sections of the manuscript. All authors contributed to manuscript revision, read and approved the submitted version.

## Conflict of Interest

The authors declare that the research was conducted in the absence of any commercial or financial relationships that could be construed as a potential conflict of interest.
